# Implementation outcomes of the WHO psychosocial intervention problem management plus in humanitarian settings: a systematic review

**DOI:** 10.1017/S2045796026100845

**Published:** 2026-07-28

**Authors:** Michela Marchetti, Federica Patania, Matilde Piccoli, Richard A. Bryant, Iryna Frankova, Marcio Gagliato, Dharani Keyan, Giuliana Mazzoni, Ana C. Molina, Marit Sijbrandij, Anke Witteveen, Orso Muneghina, Corrado Barbui, Marianna Purgato

**Affiliations:** 1 Department of Dynamic and Clinical Psychology and Health Studies, Sapienza University of Rome, Italy; 2 MHPSS Hub, SOS Children’s Villages Italy, Milan, Italy, Italy; 3 Department of Neurosciences, Biomedicine and Movement Sciences, University of Verona, Italy; 4 WHO Collaborating Centre for Research and Training in Mental Health and Service, Verona, Italy; 5 Department of Psychology, University of New South Wales, Sidney, Australia; 6 Black Dog Institute, Sydney, Australia, Australia; 7 Department of Clinical, Neuro- and Developmental Psychology, Amsterdam Public He, Netherlands; 8 ARQ Centrum 45, Oegstgeest, The Netherlands, Netherlands; 9 The Mental Health and Psychosocial Support Network – MHPSS.net, San Paolo, Brazil; 10 Fordham University, New York City, New York, USA; 11 School of Psychology, University of New South Wales, Australia; 12 Department of Clinical, Neuro- and Developmental Psychology, Vrije Universiteit Amsterdam, Amsterdam, Netherlands; 13 Global Program Expert Group on Mental Health and Psychosocial Support, SOS Children's Villages Italy, Italy

**Keywords:** humanitarian contexts, implementation outcomes, problem management plus, systematic review

## Abstract

**Aims:**

Populations affected by humanitarian crises, including conflict, disasters and displacement, are frequently exposed to elevated psychological distress, while access to mental health services remains scarce. Problem Management Plus (PM+), a low-intensity psychological intervention developed by the World Health Organization, has shown clinical efficacy. However, evidence on its implementation in humanitarian contexts remains limited. This systematic review aimed to synthesize available evidence on the implementation outcomes of PM+ and its delivery formats (individual, group and digital) in such settings. The review included individual PM+, group PM+ and Step-by-Step.

**Methods:**

Following PRISMA 2020 guidelines, we systematically searched four databases (PubMed, Scopus, Web of Science and CENTRAL) for studies published up to June 2025. Eligible studies included populations in humanitarian settings receiving PM+ in any format and reported at least one implementation outcome based on Proctor’s framework (acceptability, adoption, appropriateness, feasibility, fidelity, cost, penetration and sustainability). Data extraction and quality appraisal were conducted independently by two reviewers. The protocol for this systematic review was prospectively registered in PROSPERO (Registration No. CRD42024551943).

**Results:**

Of 2093 records screened, 23 studies met inclusion criteria, representing 5377 participants across diverse humanitarian contexts. Feasibility (70% of studies) and acceptability (65%) were the most frequently assessed outcomes, with consistently positive findings, including adequate recruitment, retention and cultural adaptability of PM+. Evidence was strongest for participant and provider acceptability and feasibility of delivery, whereas system-level outcomes such as sustainability, adoption, penetration and cost were rarely reported. Delivery by trained non-specialist providers was common and supported by supervision structures. Fidelity assessments (43%) demonstrated high adherence to intervention manuals. In contrast, sustainability (9%) and cost evaluations (17%) were infrequently reported. Barriers to implementation included stigma, population mobility and resource constraints, while facilitators included contextual adaptation, community engagement and ongoing supervision.

**Conclusions:**

PM+ demonstrates strong feasibility and acceptability when delivered by trained non-specialist providers in humanitarian contexts. However, gaps remain in evidence on long-term sustainability, cost-effectiveness and policy integration. The limited availability of system-level implementation data constrains conclusions regarding large-scale integration of PM+ in humanitarian settings. Future research should employ standardized implementation science metrics and focus on strategies to enhance scalability and embed PM+ within existing health systems.

## Background

Humanitarian settings include low- and middle-income countries (LMICs) affected by armed conflict, natural disasters, complex emergencies and forced displacement, encompassing protracted crises and urban areas hosting refugees and internally displaced persons (IDPs; Elrha, 2024). These settings present acute challenges to mental health care delivery due to systemic fragilities and overstretched services. In 2024, over 360 million people required humanitarian assistance, with over 70% living in LMICs where health systems often cannot meet basic needs (OCHA, 2023; WHO and OCHA, 2025). Among the primary factors contributing to humanitarian need is displacement, affecting over 120 million people, the majority of whom live in low-resource host communities (UNHCR, 2025).

The psychological impact of humanitarian crises is widespread and diverse. While most people demonstrate resilience, many others suffer from prolonged stress, anxiety, depression or post-traumatic symptoms, often exacerbated by social determinants such as poverty, insecurity and disrupted support networks (Ventevogel *et al.*, 2015; Charlson *et al.*, 2019; Kohrt and Kaiser, 2021). The prevalence of common mental disorders in crisis-affected populations is estimated at 22.1%, significantly higher than the 13% in non-crisis settings (Lund *et al.*, 2018; Patel *et al.*, 2018; Charlson *et al.*, 2019; WHO, 2025). Yet access to care remains critically limited, as fewer than 1 in 10 affected individuals receive the evidence-based intervention they would need (Kohrt *et al.*, 2019).

To bridge this gap, the World Health Organization (WHO) has developed a suite of different scalable, low-intensity psychological interventions. Among them, Problem Management Plus (PM+) is a brief, transdiagnostic intervention designed for delivery by non-specialists providers in low-resource and crisis-affected settings (Dawson *et al.*, 2015). PM+ has been shown to reduce psychological distress in diverse contexts such as refugee camps and conflict zones, in both individual and group formats (Schäfer *et al.*, 2023; Mwangala *et al.*, 2024; Papathanasiou *et al.*, 2024; Compri *et al.*, 2025). Digital versions like Step-by-Step (SbS) further increase accessibility (Burchert *et al.*, 2019). These interventions are essential components of global strategies to reduce the mental health treatment gap in humanitarian settings.

While several studies have confirmed the clinical efficacy of PM+, increasing attention is currently directed towards understanding how scalable psychological interventions can be implemented and scaled across different contexts, including those affected by humanitarian crises (Jordans and Kohrt, 2020; Ceccarelli *et al.*, 2024). Implementation science provides a valuable framework to understand how interventions are adopted, delivered and sustained in complex environments (Proctor *et al.*, 2011), focusing on outcomes such as acceptability, feasibility, fidelity and sustainability. Proctor et al.’s implementation outcomes framework was selected because it provides a comprehensive, multi-level taxonomy of outcomes relevant to complex service delivery contexts and has been widely applied in global mental health and implementation science. Implementation research in humanitarian settings plays a critical role in informing the feasibility and implementation of psychological interventions, offering evidence-based guidance that can support decision-making, and enable appropriate adaptations to the specific challenges and population’s needs (Ndlovu *et al.*, 2024). Humanitarian settings present implementation challenges that are distinctive from those in other settings, including severe workforce shortages, population mobility, institutional instability and reliance on short-term donor funding. These contextual factors complicate the delivery and sustainability of psychological interventions and highlight the need to examine implementation outcomes alongside clinical effectiveness (Tol *et al.*, 2023).

To date, existing reviews on PM+ have focused on its clinical effectiveness only (Schäfer *et al.*, 2023; Cai *et al.*, 2025). While evidence on the clinical effectiveness of PM+ is growing, implementation outcomes represent a distinct domain of inquiry, focusing on how interventions are adopted, delivered and sustained within real-world systems rather than on symptom reduction alone.

No systematic reviews have examined PM+ implementation outcomes, especially in humanitarian settings. Recent reviews have begun to examine implementation-related outcomes of PM+ and related interventions. For instance, Mwangala *et al.* (2024) identified key outcomes such as adaptability, acceptability, feasibility, scalability and cost-effectiveness across diverse settings. However, their scoping approach included both high- and low-income contexts and did not focus specifically on humanitarian settings. The present systematic review builds on this work by applying a rigorous methodology to synthesize implementation outcomes specifically in humanitarian contexts, thereby refining the focus of the existing evidence base.

This systematic review aimed to (a) synthesize evidence on implementation outcomes of PM+ and its variants in humanitarian settings, (b) examine differences across delivery formats (individual, group and digital) and (c) identify evidence and research gaps in system-level implementation outcomes, including adoption, penetration, sustainability and cost.

## Methods

The present systematic review was conducted following the Preferred Reporting Items for Systematic Reviews and Meta-analyses (PRISMA) 2020 guidelines for data reporting (Page *et al.*, 2021), see Supplementary Appendix A. The review protocol was prospectively registered in the PROSPERO database of systematic review protocols (Registration No. CRD42024551943).

### Search strategy

We searched four electronic databases: PubMed, Scopus, Web of Science and the Cochrane Central Register of Controlled Trials (CENTRAL). The search covered the entire available timeframe for each database up to June 2025. Keywords and phrases were combined to identify studies testing and/or implementing the Problem Management Plus (PM+) intervention, including its individual, group-based (Group PM+) and digital/self-help (SbS) versions, within humanitarian settings (e.g., crisis zones, conflict-affected areas and refugee contexts). The search strategy also included terms related to implementation outcomes (e.g., feasibility, acceptability and sustainability) and mental health (e.g., psychological well-being and scalable interventions). In addition to electronic searches, we conducted a manual review of reference lists from relevant prior reviews.

Complete search strategies for all databases are available in Supplementary Appendix B.

### Study selection and data extraction

Studies were included if they met the following criteria: (a) any study design (e.g., experimental, observational, qualitative studies and mixed-methods); (b) tested and/or implemented the PM+ intervention and its other versions; (c) were conducted in settings identified as humanitarian following Elrha’s classification (*Conflict-affected locations; Natural hazard-driven disasters; Complex emergencies; Refugee or IDP camps/settlements including in protracted crises; Refugees and IDPs in urban settings* [Elrha, 2024]); (d) assessed at least one implementation outcome, defined according to established framework by Proctor *et al.* (2011) (see Table 1 for details); (e) focused on mental health-related topics; (f) people above 16 years old (e.g., adults and young adults); (g) any control group [e.g., treatment as usual (TAU) and waiting list] or lack of control group. Studies were eligible if they reported empirical data on the implementation of PM+ or its variants in humanitarian settings. Qualitative studies were included only when they explicitly addressed implementation outcomes as defined by Proctor et al.’s framework.

**Table 1.** Implementation outcomes definitions Table 1 long description.

	Definition (Proctor *et al.*, 2011)	Stage of implementation	Type of reporting
Acceptability	The perception of stakeholders that the intervention is agreeable or satisfactory	Pre-implementation; During implementation; Post-implementation	Qualitative Quantitative
Feasibility	The extent to which an intervention can be successfully carried out	During implementation; Post-implementation	Qualitative Quantitative
Fidelity	The extent to which the intervention was implemented according to its original design	During implementation; Post implementation	Quantitative
Implementation costs	The overall cost of delivery of an intervention	Post-implementation	Quantitative
Sustainability	The maintenance of an intervention and its continued use	During implementation; Post-implementation	Quantitative Qualitative
Appropriateness	The fit, relevance or compatibility of the intervention for the setting, service provider or service user	Pre-implementation	Qualitative Quantitative
Penetration	The integration of the intervention into a routine service or a measure of how many those eligible are receiving it	During implementation; Post-implementation	Quantitative
Adoption	The process of putting an intervention to use	Pre-implementation; During implementation	Qualitative Quantitative

Screening of titles and abstracts was independently performed by two reviewers (M.M. and F.P.) using the online software *Rayyan* (Ouzzani *et al.*, 2016). Records meeting preliminary inclusion criteria were retrieved in full text screening and independently reviewed by the first two authors.

Three reviewers (M.M., F.P., and M.Pi.) then extracted key data from each eligible study, including study design, type of humanitarian setting, study aims, participant demographics (e.g., age and gender), clinical condition and implementation outcomes.

Study quality was assessed using the Mixed Methods Appraisal Tool (MMAT) (Hong *et al.*, 2018). All steps of the selection, extraction and appraisal processes were conducted independently by three reviewers (M.M., F.P., and M.Pi.). Any disagreements were addressed through discussion and resolved with the involvement of a fourth senior reviewer (M.Pu.).

### Data analysis and synthesis

Given the methodological and contextual diversity across the included studies, a narrative synthesis approach was applied to systematically summarize and interpret the findings from qualitative, quantitative and mixed-method primary studies (Popay *et al.*, 2005). Additionally, we used descriptive statistics (e.g., frequency tables) to summarize study characteristics, types of implementation outcomes evaluated and quality appraisal results. To address variability in the reporting and terminology of implementation outcomes, two reviewers (M.M. and F.P.) independently categorized the outcomes based on the Proctor framework (Proctor *et al.*, 2011), which was pre-defined. The outcomes list included: acceptability, feasibility, fidelity, implementation, costs, sustainability, appropriateness, penetration and adoption. Any disagreements were resolved through discussion and consensus, with the involvement of a third reviewer when needed, to ensure consistency and reliability in the classification process. Implementation outcomes were mapped onto Proctor et al.’s framework using predefined coding rules. Acceptability was coded as stakeholder perceptions of intervention value or satisfaction; feasibility as the practicality of delivering PM+ within existing resources and constraints; fidelity as adherence to intervention protocols; and implementation as processes related to delivery and integration. Outcomes were further classified by level of the involved stakeholders (participant, provider and organizational).

Quality appraisal was conducted using the Mixed Methods Appraisal Tool (MMAT) (Hong *et al.*, 2018), which allows for the assessment of qualitative, quantitative and mixed-methods studies, providing a consistent framework to evaluate diverse research designs within a single review.

To enhance analytical clarity, the narrative synthesis was structured along three dimensions: (i) delivery format (individual, group and digital interventions), (ii) stakeholder level (participant, provider and organizational/system) and (iii) implementation outcome domains defined by Proctor et al. This framework enabled a systematic comparison of implementation evidence across formats and levels of analysis.

## Results

Results are presented according to delivery format and stakeholder level within each implementation outcome domain. This structure was adopted to ensure coherence with Proctor’s framework and to facilitate interpretation of implementation patterns across heterogeneous contexts.

Implementation outcomes of primary studies were derived from diverse sources of evidence. Some outcomes were measured using standardized quantitative instruments, whereas others emerged from qualitative thematic analyses or bespoke assessment tools. Qualitative findings are reported as thematic evidence and should not be interpreted as directly comparable across studies. This distinction was made explicit to avoid overstating cross-study equivalence of implementation outcomes.

### Study selection

**Figure 1.**
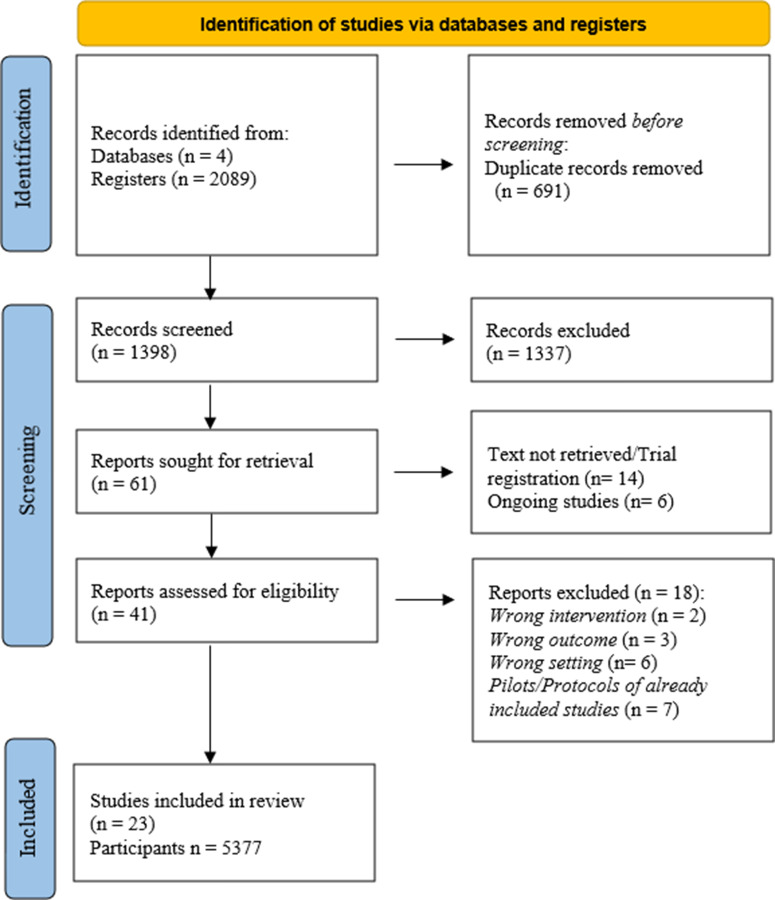
PRISMA flow-chart diagram.

A total of 2089 records were identified through the search strategy and screened at the title and abstract stage. 61 studies were eligible for full-text screening. Of these, 23 studies with 5377 participants met the inclusion criteria. The primary reasons for exclusion were wrong setting (6), wrong outcome (3) and wrong intervention (2). Furthermore, 7 studies were pilot or protocols of already included studies (Figure 1, flowchart). See Supplementary Appendix C for the full references of included and excluded studies, with reasons for exclusion.

Considerable heterogeneity was observed across included studies. The observed heterogeneity is related to (a) methodological heterogeneity: study design (randomized trials, quasi-experimental studies, qualitative studies and mixed-methods designs), (b) clinical and social heterogeneity: humanitarian setting type (conflict-affected, displacement, post-disaster and protracted crisis contexts), (c) implementation stage (pilot, trial-adjacent and early routine implementation) and (d) target populations (adults, women, refugees, IDPs and mixed community samples). Due to this heterogeneity, quantitative synthesis was not feasible (Purgato and Adams, 2012; Cumpston *et al*., 2022) however, these dimensions were systematically documented and incorporated into the narrative synthesis.

### Study characteristics

Study characteristics are summarized in Tables 2 and 3 shows, in addition, the measurement approaches and a complete table with all study details is included in the supplementary materials, Supplementary Appendix D. The 23 included studies were published between 2016 and 2025, with the majority conducted between 2022 and 2025 (14, 61%), followed by studies published from 2019 to 2021 (7, 30%). A small number of studies was published from 2016 to 2018 (2, 9%). The most commonly evaluated interventions were Problem Management Plus (PM+) alone (8, 35%), SbS (6, 26%) and PM+ in combination with other therapies (2, 9%). Group-based PM+ (gPM+) was assessed in 2 studies (9%), and gPM+ combined with other therapies in 5 studies (22%).

**Table 2.** Characteristics of included studies Table 2 long description.

	*N*	%
	23	100
*Intervention*		
PM+	8	35
PM+ and CAU or ECAU	2	9
Step-by-step	6	26
gPM+	2	9
gPM+ and CAU or ECAU	5	22
*Population*		
Adults in Care Settings	3	13
Adults with Elevated Distress	2	9
Displaced/Migrant Adults	9	39
Key Informants & Health Workers	3	13
Young adults and community	6	26
*Clinical Condition*		
Psychological distress/ Impaired functioning	3	13
Depression	4	17
PTSD	2	9
Depression, anxiety, PTSD	7	30
Depression and PTSD	2	9
N/A	5	22
*Mean age*		
18–30	7	30
31–40	9	39
41–50	5	22
N/A	2	9
*Type of humanitarian setting*		
Refugee or displacement settings	9	39
Urban/non-refugee settings	8	35
Crisis or conflict settings	6	26
*Study design*		
Mixed-methods	5	22
Qualitative study	6	26
Pragmatic Randomized Trial	2	9
RCT (cluster)	4	17
RCT (individual)	5	22
Pilot RCT	1	4
*Year*		
2016–2018	2	9
2019–2021	7	30
2022–2025	14	61
*MMAT quality rating*		
* (1 star) or 20% quality criteria met	0	0
** (2 stars) or 40% quality criteria met	4	17
*** (3 stars) or 60% quality criteria met	5	22
**** (4 stars) or 80% quality criteria met	5	22
***** (5 stars) or 100% quality criteria met	9	39

PM+: Problem Management Plus; gPM +: Group Problem Management Plus; MMAT: Mixed Methods Appraisal Tool; CAU: Enhanced Care as Usual; ECAU: Enhanced Care as Usual.

**Table 3.** Measurement approaches Table 3 long description.

Implementation outcome	Measurement approach	Type of instrument	Level of analysis	Context of assessment
Acceptability	Interviews, focus groups, satisfaction surveys	Bespoke qualitative/non-validated surveys	Participant, provider	Post-implementation
	Likert-scale questionnaires	Partially validated/study-specific	Participant	Trial contexts
Feasibility	Attendance, attrition, completion rates	Quantitative operational indicators	Participant	Trial and pilot studies
	Implementation logs, supervision reports	Bespoke operational metrics	Provider, organization	Post-implementation
Fidelity	Observation checklists, supervision tools	Study-specific structured tools	Provider	Trial-adjacent
	Competency assessments during training	Structured but non-standardized tools	Provider	Pre-implementation
Sustainability	Interviews with stakeholders	Qualitative	Organizational/system	Post-implementation
Cost	Cost-per-participant, program cost estimates	Bespoke economic analyses	System level	Trial/pilot contexts
Appropriateness	Qualitative interviews	Qualitative	Community/system	Post-implementation
Adoption/penetration	Not assessed	–	–	–

The populations targeted varied, with the largest proportion of studies focusing on displaced or migrant adults (9, 39%). Other populations included young adults and community residents (6, 26%), adults in care settings (3, 13%), key informants and health workers (3, 13%) and adults with elevated distress (2, 9%). The average age of participants across studies most commonly fell within the 31–40 age range (9, 39%), followed by ages 18–30 (7, 30%) and 41–50 (5, 22%). Participant demographics, including gender, were extracted where reported. However, gender-disaggregated implementation outcomes were inconsistently reported across studies, limiting systematic analysis. Practical problems and social determinants (e.g., unemployment, displacement and insecurity) were extracted when reported and coded as contextual implementation determinants rather than intervention outcomes.

Included studies were carried out across 10 countries: Central African Republic, Colombia, Egypt, Jordan, Kenya, Lebanon, Malawi, Nepal, Pakistan and Turkiye. All studies were conducted in humanitarian settings, particularly in refugee or displacement contexts (9, 39%), urban or non-refugee environments (8, 35%), crisis or conflict-affected settings (6, 26%).

The most common study designs included qualitative designs (6, 26%), mixed-methods designs (5, 22%) and RCTs, either individual (5, 22%) or cluster-based (4, 17%). A smaller proportion were pragmatic randomized trials (2, 9%) and 1 pilot RCT (4%).

Regarding clinical outcomes, depression, anxiety and post-traumatic stress disorder (PTSD) were the most frequently evaluated conditions, assessed in 7 studies (30%). These were followed by depression alone (4 studies, 17%) and psychological distress or impaired functioning (3 studies, 13%). Finally, PTSD alone was assessed in two studies (9%), as well as depression in comorbidity with PTSD, which was also examined in two studies (9%).

### Quality of included studies

The quality of the included studies, as assessed using the MMAT scoring system, was on average 4 out of 5. Among the included studies, 9 studies (33%) met 100% of the MMAT quality criteria (5/5), while 5 studies (22%) each met 80%, 60% (4/5 and 3/5, respectively), and 4 studies (17%) met 40% of the criteria (2/5). No studies received the lowest quality rating (1/5). Several studies showed methodological weaknesses according to the MMAT criteria. In particular, incomplete outcome data and lack of adherence to assigned interventions were common issues, affecting at least six studies. A few studies also failed to blind outcome assessors or had groups that were not comparable at baseline. Among the qualitative studies, some lacked coherence between data sources and analysis or did not sufficiently substantiate their interpretations with data. Finally, two mixed-methods studies did not adequately address divergences between qualitative and quantitative findings, nor fully meet the quality standards of each methodological tradition. See Supplementary Appendix D for the complete evaluation of all included studies.

### Implementation outcomes

Implementation outcomes are presented according to Proctor et al.’s framework. Within each outcome domain, findings are interpreted with attention to delivery format and stakeholder level, highlighting similarities and differences across implementation contexts.

Across the included studies, feasibility was the most commonly reported implementation outcome (16 studies, 70%), followed by acceptability (15 studies, 65%), fidelity (10 studies, 43%), implementation cost (4 studies, 17%), sustainability (2 studies, 9%) and appropriateness (1 study, 4%). Although penetration was pre-specified in the PROSPERO protocol, no included studies reported quantitative indicators of integration into routine services or population coverage. We therefore retained penetration as an outcome category but report it as an evidence gap.

Across included studies, women constituted the majority of participants where gender was reported, reflecting the frequent targeting of PM+ among women experiencing adversity. However, few studies explicitly examined gender-specific implementation barriers or facilitators.

**Figure 2.**
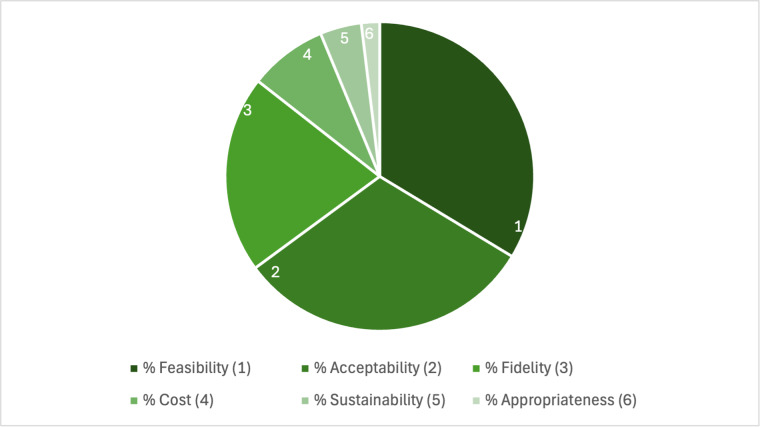
% Implementation Outcomes of the included studies.

Figure 2 and Table 4 provide an overview of the implementation outcomes and their characteristics. Figure 2 presents six of the eight outcomes, as no studies reported data for the remaining two.

**Table 4.** Overview of implementation outcomes characteristics Table 4 long description.

	Acceptability		Feasibility		Fidelity		Sustainability		Cost		Appropriateness		Adoption	
	*N*	%	*N*	%	*N*	%	*N*	%	*N*	%	*N*	%	*N*	%
	15	65	16	70	10	43	2	9	4	17	1	4	0	0
*Assessment approach*														
Qualitative	13	87			1	10	2	100			1	100		
Quantitative	1	7	16	100	9	90			4	100				
Mixed	1	7												
*Data collection method*														
Semi-structured interviews	6	40					2	100			1	100		
Focus group discussions	2	13			1	10								
Survey/Checklist	1	7			7	70								
Attendance/attrition rates			15	94										
Supervision					2	20								
Other/combined	6	40	1	6	4	40			4	100				
*Population reporting*														
Participants	5	33	8	50										
Stakeholders	4	27	1	6										
Interventionist			2	13			1	50						
Family/Community											1	100		
Mixed (combinations of the above)	6	40	4	25			1	50						
Independent rater					10	100								
*Assessment timeline*														
Pre-implementation	1	7	2	13	9	90								
During implementation	2	13												
Post-implementation	12	80	14	88	1	10	2	100	4	100				

#### Acceptability

Evidence on acceptability was primarily reported at the participant and provider levels, with variations observed across individual, group and digital delivery formats. Acceptability was assessed using a combination of validated quantitative instruments and bespoke qualitative or survey-based approaches, which limits direct comparability across studies.

Acceptability was assessed in 65% of studies, predominantly through qualitative methodologies (13, 87%), with minimal use of quantitative (1, 7%) or mixed-methods (1, 7%) approaches. Data collection was mostly conducted through semi-structured interviews (6; 40%), while some studies assessed this outcome through focus group discussions (2, 13%), surveys (1, 7%) and combined methods (6, 40%). The majority of acceptability assessments occurred at post-implementation (12, 80%) and were reported by different respondent groups, including participants (5, 33%), stakeholders (4, 27%) and a combination of participants, stakeholders and interventionists (6, 40%).

Findings indicate PM+ and gPM+ were consistently regarded as acceptable, relevant and beneficial, across studies (Van’t Hof *et al.*, 2018; Heim *et al.*, 2021; Acarturk *et al.*, 2022; Abi Ramia *et al.*, 2023; Hussain *et al.*, 2025; Mwale *et al.*, 2025). Participants frequently highlighted the usefulness of the four core strategies, stress management, behavioural activation, problem-solving and strengthening social support, which they reported applying to manage daily stressors and family relationships (Dozio *et al.*, 2021; Greene *et al.*, 2024; Miller-Suchet *et al.*, 2024). In quantitative assessments, 77% of participants indicated that they had learned new knowledge, 63% reported that the sessions provided opportunities to meet others, and 45% found that sessions allowed them to relax. Qualitative data further illustrated perceived mechanisms of change: participants described improved communication, patience and anger regulation, as well as reductions in somatic symptoms such as restlessness and weakness (Greene *et al.*, 2024; Miller-Suchet *et al.*, 2024). Both male and female participants emphasized the value of the group format, which was described as fostering a sense of shared experience and providing a safe space to disclose intimate or distressing problems (Sangraula *et al.*, 2020; Acarturk *et al.*, 2022; Perera *et al.*, 2022). Non-specialist providers and mentors also reported personal benefits from delivering the intervention, including increased knowledge about stress, enhanced confidence in managing their own problems and improved general well-being (Van’t Hof *et al.*, 2018; Kangwana *et al.*, 2024). At the same time, several barriers to engagement were reported, including illness, family and childcare responsibilities, competing obligations, logistical difficulties (e.g., extreme weather) and stigma (Heim *et al.*, 2021; Greene *et al.*, 2024). In particular, limited acceptance of psychological help, especially in app-based formats, was identified among Syrian refugees as a key barrier (Burchert *et al.*, 2019).

Overall, the interventions were positively evaluated across respondent groups, with participants expressing satisfaction and willingness to recommend or re-engage with the programme. In one study, more than 90% of participants reported being satisfied and 92% stated they would return to the programme if they were to seek help again.

#### Feasibility

Feasibility findings predominantly reflected participant-level and provider-level indicators across delivery formats, while system-level feasibility was rarely assessed. Measurement approaches included standardized quantitative indicators (e.g., retention, attendance and completion rates) as well as study-specific operational metrics and qualitative assessments, highlighting methodological heterogeneity across studies.

Feasibility was assessed in 70% of included studies, all of which employed quantitative methods (16, 100%). The most prevalent data source was attendance and attrition records (15, 94%), supplemented by combined methods (1, 6%). The majority of feasibility assessments were conducted at post-implementation (14, 88%), while a smaller proportion were conducted pre-implementation (2, 13%). Reporting groups included participants (8, 50%), interventionists (2, 13%), stakeholders (1, 6%) and mixed groups (4, 25%).

Feasibility outcomes varied across studies but generally supported the possibility of implementing PM+ and gPM+. Intervention delivery by non-specialists [e.g., Community Health Workers (CHWs) and non-specialist providers] was feasible following brief training and supervision (Dawson *et al.*, 2016; Van’t Hof *et al.*, 2018; Kangwana *et al.*, 2024). Recruitment rates and intervention implementation were adequate in most settings, with engagement and adherence indicating general acceptance. Retention and completion rates differed by setting and study condition. In several trials, approximately two-thirds of participants attended three or more sessions, with completion often exceeding 70% among those who initiated the intervention (Khan *et al.*, 2019; Akhtar *et al.*, 2021; Mwale *et al.*, 2025). Retention was notably higher in specialized support conditions compared to non-specialized arms. Some studies reported very low drop-out rates (e.g., <6%) (Perera *et al.*, 2022), while others noted high attrition due to contextual factors such as mobility, competing responsibilities and technical barriers (Abi Ramia et al., 2023; Burchert *et al.*, 2024). Despite variability, several trials reported high follow-up completion (up to 97.5%) and low refusal rates, indicating both feasibility and participant commitment. Group formats demonstrated consistent feasibility across genders and regions, with qualitative findings supporting cultural and logistical acceptability (Khan *et al.*, 2019; Sangraula *et al.*, 2020; Akhtar *et al.*, 2021).

#### Fidelity

Fidelity was mainly assessed at the provider level and in trial-adjacent contexts, with limited evidence from routine implementation settings. Most fidelity assessments relied on supervision checklists, structured observation tools or training-related evaluations, rather than longitudinal monitoring during real-world service delivery. Therefore, findings should not be interpreted as evidence of stable fidelity over time or across implementation phases.

Fidelity was assessed in 43% of studies, using for the majority quantitative methodologies (9, 90%) and one study used qualitative methodology with focus group discussion (1, 10%). Quantitative tools included structured surveys or checklists (7, 70%), supervision records (2, 20%) and combined sources (4, 40%). Fidelity assessments were uniformly conducted by independent raters (10, 100%), with data collection occurring primarily at the pre-implementation stage (9, 90%).

Findings indicate that non-specialist providers, including Community Health Volunteers (CHVs) and CHWs, demonstrated competent delivery of PM+ following brief training and supervision. Mentors adhered well to core intervention principles. Fidelity was higher in the nonspecialized support condition (78%) than in the specialized support condition (64%; *p* = 0.037) (Greene *et al.*, 2024). Group PM+ facilitators scored ≥75% fidelity across sessions (Sangraula *et al.*, 2020). Minor protocol deviations occurred in 6% of sessions (Cuijpers *et al.*, 2022). Overall, 80% of core components were delivered as intended in fidelity assessments.

#### Sustainability

Evidence on sustainability was scarce and primarily derived from qualitative data at the organizational and system levels. The limited number of studies addressing sustainability does not indicate low relevance of this outcome, but rather a significant evidence gap in the current implementation literature.

Sustainability was explored in 2 studies (9%), all of which adopted qualitative approaches and used semi-structured interviews for data collection. These assessments were conducted exclusively post-implementation. CHVs’ integration within Kenya’s primary healthcare system supports sustainable PM+ delivery, though limited funding for human resources poses a significant barrier (Van’t Hof *et al.*, 2018). Challenges remain around CHV selection, training, supervision and compensation. Both facilitators and participants expressed strong interest in continuing gPM+ post-study (Greene *et al.*, 2024), emphasizing the need for formal certification and organizational support to enhance programme acceptance and sustainability.

#### Implementation costs

Evidence on implementation costs was limited and heterogeneous, with studies employing bespoke costing approaches rather than standardized economic evaluation frameworks. The scarcity of cost data represents a critical gap for informing scale-up decisions in humanitarian contexts.

Implementation costs were assessed in two studies (9%). We identified substantial variation in costs across interventions and implementation models, with differences observed in programme expenditures, per-user costs and economic efficiency (Hamdani *et al.*, 2020; Abi Hana *et al.*, 2024; Greene *et al.*, 2024; McBain *et al.*, 2024). Costs were disaggregated by intervention type, participant and supervisory model, indicating greater cost-effectiveness for approaches utilizing local trainers and integrated care models compared to standard alternatives.

#### Appropriateness

Appropriateness was rarely assessed and relied exclusively on qualitative evidence, indicating a substantial gap in systematic measurement of contextual fit across delivery formats and stakeholder levels.

Appropriateness was assessed qualitatively in only one study finding that, during the implementation of PM+, community engagement improved the cultural relevance and contextual fit of the intervention (Nakkash *et al.*, 2024).

#### Adoption

No included studies explicitly measured adoption or penetration using standardized indicators at the organizational or system level. The absence of evidence on these outcomes should be interpreted as a major implementation research gap rather than a lack of relevance for PM+ scale-up.

## Discussion

To the best of our knowledge, this is the first systematic review to summarize the implementation outcomes of the WHO PM+ intervention and its variants in humanitarian contexts. We reviewed 23 studies involving 5377 participants. Included studies adopted different research designs, such as qualitative, quantitative (i.e., cluster or individual RCT) and mixed-methods studies. The implementation metrics varied according to the study design, as they reflected the methodology adopted in the primary study. Our systematic review provides compelling evidence that PM+ and its variants are largely feasible, acceptable and implementable across different types of humanitarian settings.

From the included studies, acceptability emerged as one of the most consistently reported implementation outcomes. PM+ was widely perceived as relevant, beneficial and responsive to local needs by both participants and non-specialist providers. Group-based formats were particularly valued for fostering social support and collective coping, in line with broader evidence on the protective role of social connectedness during adversity (Hobfoll *et al.*, 2007; Schäfer *et al.*, 2024). Acceptability was further enhanced through contextual adaptations, such as integrating culturally resonant metaphors and adjusting delivery schedules, consistent with evidence that cultural tailoring improves engagement and outcomes in low-resource settings (Heim and Kohrt, 2019). Evidence on digital PM+ delivery remains limited but suggests distinct acceptability challenges compared to face-to-face formats. For example, Burchert *et al.* (2019) reported hesitancy towards app-based psychological support among Syrian participants, reflecting concerns about stigma, trust and technological access. These findings align with those of other qualitative and quantitative studies in the field of global and public mental health (Purgato *et al.*, 2024; Sculco *et al.*, 2025). They highlight the need to differentiate implementation dynamics and strategies across individual, group and delivery formats (i.e., digital versus in-person). Barriers and facilitators appear to operate differently across delivery formats. In individual PM+, barriers were often related to stigma, confidentiality concerns, and competing caregiving responsibilities. In group formats, mobility constraints and gender norms sometimes affected attendance, while peer support functioned as a key facilitator. In digital delivery models, technical access, digital literacy and trust in app-based platforms emerged as primary barriers, suggesting that implementation strategies must be format-specific rather than universally applied.

Findings on feasibility strongly support the delivery of PM+ by non-specialist providers following brief training and structured supervision, in line with WHO’s task-shifting strategy (van Ginneken *et al.*, 2013; Mendenhall *et al.*, 2014; Singla *et al.*, 2017). High retention and low refusal rates in most studies may reflect robust community engagement, although attrition due to displacement, stigma or competing demands and priorities remains a significant challenge. Importantly, supervision is a critical determinant of implementation success (Abujaber *et al.*, 2022; Chutiyami *et al.*, 2025). Evidence on fidelity, though limited, suggests promising adherence to PM+ protocols and adequate competency among non-specialist providers, with some studies reporting higher fidelity scores in non-specialist versus specialist conditions. Taken together, the evidence suggests that successful implementation of PM+ in humanitarian settings depends on a set of recurring enabling conditions. These include (i) structured initial training combined with ongoing supervision and competency monitoring; (ii) meaningful community engagement to enhance trust and reduce stigma; (iii) context-sensitive cultural adaptation processes and (iv) integration within existing service delivery platforms to support sustainability. These conditions appear to function as core implementation requirements rather than optional enhancements, and should be considered essential components of scale-up strategies.

This finding supports the robustness and scalability of training models designed for task-shifted interventions (Purgato *et al.*, 2025). However, most fidelity assessments were conducted pre-implementation, leaving crucial gaps in understanding how fidelity evolves over longer periods of time or across different phases of humanitarian response (e.g., acute emergencies versus protracted displacement). Incorporating the Ensuring Quality in Psychological Support (EQUIP) (Kohrt *et al.*, 2020) tools into the implementation process would provide a structured means of assessing whether fidelity to the protocol and competency would be sustained over time. Data on sustainability, cost, appropriateness and adoption remain scarce or inexistent. The limited use of standardized implementation science measures further constrains cross-study comparability. Future research should adopt validated instruments to assess acceptability [e.g., standardized acceptability scales such as the Acceptability of Intervention Measure (AIM; Weiner *et al.*, 2017)], feasibility indicators beyond attendance metrics, structured fidelity monitoring tools, costing frameworks aligned with health economic standards, and sustainability indicators capturing long-term integration into health systems. Establishing common and minimal reporting standards for implementation outcomes would substantially strengthen the evidence base and support more informed scale-up decisions.

Findings suggest that institutionalization of PM+ is constrained by inconsistent funding, absence of certification pathways for non-specialist providers, and insufficient policy integration, echoing broader trends in humanitarian implementation research (Barnett *et al.*, 2018; Ndlovu *et al.*, 2024). Preliminary cost analyses nonetheless indicate potential economic efficiency when interventions leverage local trainers and integrate PM+ into existing service structures, a crucial insight for donors and policymakers seeking scalable solutions under resource constraints. Our findings align with those of Mwangala *et al.* (2024), particularly regarding the prominence of acceptability and feasibility outcomes. However, our review highlights additional gaps in system-level outcomes in humanitarian settings, such as sustainability, penetration and costs. This suggests that while PM+ is often feasible and acceptable at the participant and provider levels, evidence on integration into routine health and/or social services remains limited.

Gender emerged as an important but under-analysed dimension of PM+ implementation. Studies reported barriers such as caregiving responsibilities, confidentiality concerns and gender norms affecting participation, particularly among women (e.g., Khan *et al.*, 2019). Prior research has also highlighted challenges related to gender-matching between providers and participants and the intersection of gender with experiences of violence and social disadvantage. The limited reporting of gender-disaggregated implementation outcomes represents a significant gap in the evidence base and warrants systematic attention in future implementation research. For example, an Individual Participant Data meta-analysis should be conducted as a next step, to assess if and how much gender impacts implementation and clinical outcomes.

Our focus on implementation reflects a growing consensus in global mental health research that advancing beyond clinical efficacy is essential to achieve real-world impact, also in the long term. Previous reviews have primarily focused on clinical efficacy (Schäfer *et al.*, 2023; Papola *et al.*, 2024; Cai *et al.*, 2025), whereas this review shifts the lens towards the practical realities of delivering and sustaining PM+ in settings affected by conflict, displacement, and fragile health systems. This shift is critical, as implementation science emphasizes that clinical efficacy, despite being essential when a new intervention is developed, is insufficient for large-scale impact in real-world conditions (Aarons *et al.*, 2011; Proctor *et al.*, 2011). A focus on implementation allows the integration of psychological interventions, including those developed by the WHO, into local health systems to ensure their sustainable implementation and scale-up across settings (Purgato *et al.*, 2024). Accordingly, Papola *et al.* (2024) highlighted the potential of psychological and social interventions to address mental health needs in humanitarian crises, while also emphasizing the persistent gap between evidence generation and practical implementation. For example, the successful scale-up of interventions such as PM+ is critically influenced by barriers and facilitators, including unstable funding, limited workforce capacity and cultural adaptation needs (Troup *et al.*, 2021). By systematically analysing implementation outcomes through a standardized theoretical framework (Proctor *et al.*, 2011), this review accounted for the critical role of specific contextual barriers, such as population mobility, limited resources and sociocultural heterogeneity, that also historically hindered implementation in humanitarian settings (Tol *et al.*, 2015; Ventevogel *et al.*, 2015). Until now, these challenges had not been comprehensively reviewed for PM+, leaving policymakers and implementers without synthesized evidence to guide scaling strategies.

Several limitations of our study should be acknowledged. First, our review included only studies on PM+ interventions; future research should examine and compare implementation outcomes for other psychosocial approaches. Although PM+, group PM+ and SbS share a common theoretical foundation, they represent distinct delivery models with potentially different implementation dynamics. Even though our review is systematic and comprehensive, we recognize that synthesizing PM+ interventions within a single review may obscure format-specific patterns of implementation and should be interpreted with caution. Secondly, the scope was limited to humanitarian settings in LMICs, because these settings present specific implementation challenges and barriers. However, emergencies and context-specific challenges may also arise in high-income countries. Beyond heterogeneity across studies, several additional methodological limitations should be acknowledged. These include potential publication bias despite the comprehensive search and regular contacts with WHO Headquarters, possible language restrictions and the limited geographical and contextual scope of included humanitarian settings. Together, these factors may have influenced the availability, diversity and representativeness of the evidence base. Thirdly, we did not conduct a meta-analysis due to the heterogeneity of study designs and outcomes, and the predominance of qualitative data. Fourthly, we recognize that the majority of primary studies included in this review were not implementation studies that used strict implementation science metrics. Furthermore, much of the included evidence derives from trial-adjacent or pilot studies with specific research findings rather than routine service delivery integrated in the health systems activity. Consequently, there is limited evidence on embedded implementation within humanitarian health systems (i.e. penetration), restricting conclusions about long-term sustainability, adoption and system-level integration of PM+ interventions.

This review did not include grey literature such as NGO reports, which may contain relevant implementation insights from humanitarian settings.

Despite these limitations, this review provides a comprehensive synthesis of PM+ implementation outcomes and offers actionable insights for scale-up. The findings strongly support community-based, task-shifted delivery models as both feasible and acceptable across diverse settings, while highlighting the need for policy-level commitment to ensure sustainability. Integrating PM+ into national health policies, workforce strategies and funding mechanisms is key to bridging the mental health treatment gap in crisis-affected populations (WHO, 2023). Future research should prioritize standardized methodologies, long-term outcome data and cross-context comparisons, including high-income humanitarian settings, to enhance the generalizability and robustness of evidence. Addressing these gaps will be critical to advancing the real-world impact of scalable psychological interventions such as PM+.

PM+ holds substantial promise as a scalable intervention for mental health care in humanitarian settings. Realizing its full potential will require coordinated and continuous investments in implementation research, capacity-building and system-level integration. By structuring the synthesis across delivery formats, stakeholder levels and implementation outcome domains, the review moves beyond descriptive reporting and offers a framework-based interpretation of PM+ implementation evidence in humanitarian settings. Beyond demonstrating feasibility and acceptability, this review identifies critical structural gaps in the implementation evidence base, particularly at the system level. While PM+ appears implementable under supervised and trial-adjacent conditions, evidence on embedded delivery within routine humanitarian health systems remains limited. Advancing from pilot feasibility towards sustainable scale-up will require investment in supervision infrastructures, policy integration, workforce certification pathways and long-term funding mechanisms. By identifying both enabling conditions and systemic gaps, this review provides a systematic and implementation-oriented roadmap for strengthening the scalability of PM+ and related interventions in humanitarian contexts.

## Conclusions

This review demonstrates that WHO’s PM+ and its variants are generally feasible, acceptable and implementable in diverse humanitarian settings. Positive participant engagement, adherence to core components and the effectiveness of task-shifted delivery highlight PM+ as a promising intervention to address mental health needs in conflict-affected and displaced populations. Despite these strengths, evidence on sustainability, costs, appropriateness and adoption at organizational or system levels remains limited, constraining conclusions about long-term scale-up.

Future research should prioritize standardized assessment of all implementation outcomes, long-term follow-up, cost-effectiveness analyses and cross-context comparisons to strengthen the evidence base and guide scalable, context-appropriate mental health interventions in humanitarian settings. Based on our findings, several implementation priorities emerge. First, strengthening supervision structures and community engagement appears critical for sustaining feasibility and fidelity. Secondly, standardized training and competency-based frameworks, such as the WHO EQUIP tool and other WHO-aligned training packages, should be integrated into PM+ implementation models across settings. Finally, future research should adopt common and standardized implementation metrics, including costing frameworks and sustainability indicators, to support evidence-informed scale-up in humanitarian settings.

## Supporting information

10.1017/S2045796026100845.sm001 Marchetti et al. supplementary material Marchetti et al. supplementary material

## Data Availability

The data will be made available upon reasonable request from the corresponding author.

## Supplementary material

The supplementary material for this article can be found at https://doi.org/10.1017/S2045796026100845.

## Table 1 Long description

The flowchart outlines the process of identifying and screening studies via databases and registers. It begins with the ′Identification′ phase, where records are identified from databases (4) and registers (2089), totaling 2089 records. Before screening, 691 duplicate records are removed. In the ′Screening′ phase, 1398 records are screened, with 1337 records excluded. Reports sought for retrieval number 61, with 14 not retrieved due to trial registration and 6 ongoing studies. Reports assessed for eligibility total 41, with 18 reports excluded for reasons such as wrong intervention (2), wrong outcome (3), wrong setting (6) and pilot/protocols of already included studies (7). Finally, in the ′Included′ phase, 23 studies are included in the review, involving 5377 participants.

Navigate back to Table 1.

## Table 2 Long description

Characteristics are summarized for 23 included studies, reporting counts and percentages across intervention type, population, clinical condition, mean age, humanitarian setting, study design, publication year, and MMAT quality rating. Interventions most often involved Problem Management Plus at 35% or Step-by-Step at 26%, while group Problem Management Plus combined with care as usual variants accounted for 22%. Populations most frequently studied were displaced or migrant adults at 39%, followed by young adults and community samples at 26%; adults in care settings and key informants or health workers were each 13%. Clinical focus most commonly combined depression, anxiety, and post-traumatic stress disorder at 30%, while 22% did not specify a clinical condition. Mean age was most often 31 to 40 years at 39%, then 18 to 30 years at 30%, with 9% not reporting age. Settings were mainly refugee or displacement contexts at 39% and urban or non-refugee contexts at 35%, with crisis or conflict settings at 26%. Study designs were largely qualitative at 26% or mixed-methods and individual randomized controlled trials at 22% each; cluster randomized controlled trials were 17%, and pilot randomized controlled trials were 4%. Most studies were published in 2022 to 2025 at 61%, and MMAT ratings clustered toward higher quality, with 39% meeting all criteria and none in the lowest category.

Navigate back to Table 2.

## Table 4 Long description

Implementation outcomes are summarized by outcome type, assessment approach, data collection method, population reporting, and assessment timeline, using counts and percentages. Overall reporting was highest for feasibility at 16 studies (70 percent) and acceptability at 15 studies (65 percent), followed by fidelity at 10 studies (43 percent). Sustainability was reported in 2 studies (9 percent), cost in 4 studies (17 percent), appropriateness in 1 study (4 percent), and adoption in none. Assessment approach varied by outcome: acceptability was mainly qualitative (13 of 15, 87 percent), feasibility was entirely quantitative (16 of 16, 100 percent), and fidelity was mostly quantitative (9 of 10, 90 percent) with one qualitative study. Data collection methods differed: feasibility relied largely on attendance or attrition rates (15 of 16, 94 percent), fidelity commonly used surveys or checklists (7 of 10, 70 percent), and acceptability used semi-structured interviews and other or combined methods (each 6 of 15, 40 percent). Population reporting was mixed, with acceptability often using mixed respondent groups (6 of 15, 40 percent) and feasibility most often reporting participants (8 of 16, 50 percent); fidelity was rated by independent raters in all fidelity studies (10 of 10, 100 percent). Timing clustered after implementation, especially for acceptability (12 of 15, 80 percent) and feasibility (14 of 16, 88 percent), while fidelity was mostly assessed pre-implementation (9 of 10, 90 percent). Blank cells indicate categories not reported for that outcome, so comparisons across outcomes should be interpreted as differences in reporting rather than direct performance differences.

Navigate back to Table 4.

## Table 3 Long description

Implementation outcomes are paired with measurement approaches, instrument types, levels of analysis, and when assessment occurs. Acceptability is measured mainly through interviews, focus groups, satisfaction surveys, and Likert-scale questionnaires, using bespoke or partially validated instruments at the participant or provider level, typically after implementation or in trial settings. Feasibility uses attendance, attrition, and completion rates plus implementation logs and supervision reports, relying on quantitative operational indicators and bespoke metrics at participant, provider, or organizational levels in trials, pilots, or post-implementation. Fidelity is assessed with observation checklists and supervision tools in trial-adjacent contexts, and competency assessments during training occur before implementation using structured but non-standardized tools. Sustainability and appropriateness are assessed post-implementation through qualitative stakeholder or community interviews at organizational, system, or community levels. Cost is estimated in trials or pilots using bespoke economic analyses at the system level, such as cost per participant and program cost estimates. Adoption or penetration is listed as not assessed, indicating a gap in measurement coverage.

Navigate back to Table 3.
