# Supplementary material for: Implementation outcomes of the WHO psychosocial intervention problem management plus in humanitarian settings: a systematic review
Source: Epidemiol Psychiatr Sci. 2026 Jul 28;35:e44. doi: 10.1017/S2045796026100845 (PMC13420161; doi:10.1017/S2045796026100845)
Supplement: Marchetti et al. supplementary material [file S2045796026100845sup001.pdf]

Supplementary Materials

1. Appendix A: PRISMA 2020 Checklist

Section and Topic	Item #	Checklist item	Location where item is reported
**TITLE**			
Title	1	Identify the report as a systematic review.	Title
**ABSTRACT**			
Abstract	2	See the PRISMA 2020 for Abstracts checklist.	
**INTRODUCTION**			
Rationale	3	Describe the rationale for the review in the context of existing knowledge.	Introduction<br>(second to last paragraph)
Objectives	4	Provide an explicit statement of the objective(s) or question(s) the review addresses.	Introduction<br>(last paragraph)
**METHODS**			
Eligibility criteria	5	Specify the inclusion and exclusion criteria for the review and how studies were grouped for the syntheses.	Method (study selection)
Information sources	6	Specify all databases, registers, websites, organisations, reference lists and other sources searched or consulted to identify studies. Specify the date when each source was last searched or consulted.	Method (search strategy)
Search strategy	7	Present the full search strategies for all databases, registers and websites, including any filters and limits used.	Method (search strategy)
Selection process	8	Specify the methods used to decide whether a study met the inclusion criteria of the review, including how many reviewers screened each record and each report retrieved, whether they worked independently, and if applicable, details of automation tools used in the process.	Method (study selection and data extraction)
Data collection process	9	Specify the methods used to collect data from reports, including how many reviewers collected data from each report, whether they worked independently, any processes for obtaining or confirming data from study investigators, and if applicable, details of automation tools used in the process.	Method (study selection and data extraction)
Data items	10a	List and define all outcomes for which data were sought. Specify whether all results that were compatible with each outcome domain in each study were sought (e.g. for all measures, time points, analyses), and if not, the methods used to decide which results to collect.	Method (study selection)
	10b	List and define all other variables for which data were sought (e.g. participant and intervention characteristics, funding sources). Describe any assumptions made about any missing or unclear information.	Method (study selection and data extraction)
Study risk of bias assessment	11	Specify the methods used to assess risk of bias in the included studies, including details of the tool(s) used, how many reviewers assessed each study and whether they worked independently, and if applicable, details of	Method (study selection and data extraction)

Section and Topic	Item #	Checklist item	Location where item is reported
		automation tools used in the process.	
Effect measures	12	Specify for each outcome the effect measure(s) (e.g. risk ratio, mean difference) used in the synthesis or presentation of results.	NA
Synthesis methods	13a	Describe the processes used to decide which studies were eligible for each synthesis (e.g. tabulating the study intervention characteristics and comparing against the planned groups for each synthesis (item #5)).	NA
	13b	Describe any methods required to prepare the data for presentation or synthesis, such as handling of missing summary statistics, or data conversions.	NA
	13c	Describe any methods used to tabulate or visually display results of individual studies and syntheses.	Method (data analysis and synthesis)
	13d	Describe any methods used to synthesize results and provide a rationale for the choice(s). If meta-analysis was performed, describe the model(s), method(s) to identify the presence and extent of statistical heterogeneity, and software package(s) used.	Method (data analysis and synthesis)
	13e	Describe any methods used to explore possible causes of heterogeneity among study results (e.g. subgroup analysis, meta-regression).	NA
	13f	Describe any sensitivity analyses conducted to assess robustness of the synthesized results.	NA
Reporting bias assessment	14	Describe any methods used to assess risk of bias due to missing results in a synthesis (arising from reporting biases).	NA
Certainty assessment	15	Describe any methods used to assess certainty (or confidence) in the body of evidence for an outcome.	NA
**RESULTS**			
Study selection	16a	Describe the results of the search and selection process, from the number of records identified in the search to the number of studies included in the review, ideally using a flow diagram.	
	16b	Cite studies that might appear to meet the inclusion criteria, but which were excluded, and explain why they were excluded.	
Study characteristics	17	Cite each included study and present its characteristics.	
Risk of bias in studies	18	Present assessments of risk of bias for each included study.	
Results of individual studies	19	For all outcomes, present, for each study: (a) summary statistics for each group (where appropriate) and (b) an effect estimate and its precision (e.g. confidence/credible interval), ideally using structured tables or plots.	*NA*
Results of syntheses	20a	For each synthesis, briefly summarise the characteristics and risk of bias among contributing studies.	*NA*
	20b	Present results of all statistical syntheses conducted. If meta-analysis was done, present for each the summary estimate and its precision (e.g. confidence/credible interval) and measures of statistical heterogeneity. If	NA

Section and Topic	Ite m #	Checklist item	Location where item is reported
		comparing groups, describe the direction of the effect.	
	20c	Present results of all investigations of possible causes of heterogeneity among study results.	NA
	20d	Present results of all sensitivity analyses conducted to assess the robustness of the synthesized results.	NA
Reporting biases	21	Present assessments of risk of bias due to missing results (arising from reporting biases) for each synthesis assessed.	NA
Certainty of evidence	22	Present assessments of certainty (or confidence) in the body of evidence for each outcome assessed.	NA
**DISCUSSION**			
Discussion	23a	Provide a general interpretation of the results in the context of other evidence.	
	23b	Discuss any limitations of the evidence included in the review.	
	23c	Discuss any limitations of the review processes used.	
	23d	Discuss implications of the results for practice, policy, and future research.	
**OTHER INFORMATION**			
Registration and protocol	24a	Provide registration information for the review, including register name and registration number, or state that the review was not registered.	Methods (first paragraph)
	24b	Indicate where the review protocol can be accessed, or state that a protocol was not prepared.	Methods (first paragraph)
	24c	Describe and explain any amendments to information provided at registration or in the protocol.	NA
Support	25	Describe sources of financial or non-financial support for the review, and the role of the funders or sponsors in the review.	See financial statement
Competing interests	26	Declare any competing interests of review authors.	See declaration of interest statement
Availability of data, code and other materials	27	Report which of the following are publicly available and where they can be found: template data collection forms; data extracted from included studies; data used for all analyses; analytic code; any other materials used in the review.	See references to Appendices across the manuscript

From: Page MJ, McKenzie JE, Bossuyt PM, Boutron I, Hoffmann TC, Mulrow CD, et al. The PRISMA 2020 statement: an updated guideline for reporting systematic reviews. BMJ 2021;372:n71. doi: 10.1136/bmj.n71

2. Appendix B: Search strategy

Line 1 ("problem management plus" OR "step-by-step" OR "group problem management plus” OR “PM+” OR “gPM+”)

AND

Line 2 (“acceptability” OR “acceptable” OR “feasibility” OR “feasible” OR “fidelity” OR “adherent” OR “adherence” OR “accuracy” OR “accurate” OR "implementation cost*" OR “cost” OR “economic*” OR “expense” OR “expensive” OR “expenditure” OR “expenditures” OR “pay” OR “paid” OR “price” OR “budget” OR “sustainability” OR “sustained” OR “sustainable” OR “appropriateness” OR “appropriate” OR “suitable” OR “suitability” OR “suited” OR “relevance” OR “relevant” OR “penetration” OR “penetrated” OR “diffusion” OR “adoption” OR “adopted” OR “implementation science” OR “implementation research” OR “dissemination research” OR “implementation outcom*”)

AND

Line 3 (“mental health” OR “wellbeing” OR “psychosocial wellbeing” OR “mental health intervention” OR “scalable intervention” OR “psychological”)

a. WEB OF SCIENCE

((AB=("problem management plus" OR "step-by-step" OR "group problem management plus” OR “PM+” OR “gPM+”)) AND AB=(“acceptability” OR “acceptable” OR “feasibility” OR “feasible” OR “fidelity” OR “adherent” OR “adherence” OR “accuracy” OR “accurate” OR "implementation cost*" OR “cost” OR “economic*” OR “expense” OR “expensive” OR “expenditure” OR “expenditures” OR “pay” OR “paid” OR “price” OR “budget” OR “sustainability” OR “sustained” OR “sustainable” OR “appropriateness” OR “appropriate” OR “suitable” OR “suitability” OR “suited” OR “relevance” OR “relevant” OR “penetration” OR “penetrated” OR “diffusion” OR “adoption” OR “adopted” OR “implementation science” OR “implementation research” OR “dissemination research” OR “implementation outcom*”)) AND AB=(“mental health” OR “wellbeing” OR “psychosocial wellbeing” OR “mental health intervention” OR “scalable intervention” OR “psychological”)

b. PUBMED

(("problem management plus"[Title/Abstract] OR "step-by-step"[Title/Abstract] OR "group problem management plus"[Title/Abstract] OR "PM+"[Title/Abstract] OR "gPM+"[Title/Abstract]) AND ("acceptability"[Title/Abstract] OR "acceptable"[Title/Abstract] OR "feasibility"[Title/Abstract] OR "feasible"[Title/Abstract] OR "fidelity"[Title/Abstract] OR "adherent"[Title/Abstract] OR "adherence"[Title/Abstract] OR "accuracy"[Title/Abstract] OR "accurate"[Title/Abstract] OR "implementation cost*"[Title/Abstract] OR "cost"[Title/Abstract] OR "economic*"[Title/Abstract] OR "expense"[Title/Abstract] OR "expensive"[Title/Abstract] OR "expenditure"[Title/Abstract] OR "expenditures"[Title/Abstract] OR "pay"[Title/Abstract] OR "paid"[Title/Abstract] OR "price"[Title/Abstract] OR "budget"[Title/Abstract] OR "sustainability"[Title/Abstract] OR "sustained"[Title/Abstract] OR "sustainable"[Title/Abstract] OR "appropriateness"[Title/Abstract] OR "appropriate"[Title/Abstract] OR "suitable"[Title/Abstract] OR "suitability"[Title/Abstract] OR "suited"[Title/Abstract] OR "relevance"[Title/Abstract] OR "relevant"[Title/Abstract] OR "penetration"[Title/Abstract] OR "penetrated"[Title/Abstract] OR "diffusion"[Title/Abstract] OR "adoption"[Title/Abstract] OR "adopted"[Title/Abstract] OR "implementation science"[Title/Abstract] OR "implementation research"[Title/Abstract] OR "dissemination research"[Title/Abstract] OR "implementation outcom*"[Title/Abstract])) AND ("mental health"[Title/Abstract] OR "wellbeing"[Title/Abstract] OR "psychosocial wellbeing"[Title/Abstract] OR "mental health intervention"[Title/Abstract] OR "scalable intervention"[Title/Abstract] OR "psychological"[Title/Abstract])

c. CENTRAL

#1: "problem management plus":ti,ab,kw OR "step-by-step":ti,ab,kw OR "group problem management plus":ti,ab,kw OR "PM+":ti,ab,kw OR "gPM+":ti,ab,kw

#2: acceptability:ti,ab,kw OR acceptable:ti,ab,kw OR feasibility:ti,ab,kw OR feasible:ti,ab,kw OR fidelity:ti,ab,kw OR adherent:ti,ab,kw OR adherence:ti,ab,kw OR accuracy:ti,ab,kw OR accurate:ti,ab,kw OR (implementation NEXT cost*):ti,ab,kw OR cost:ti,ab,kw OR economic*:ti,ab,kw OR expense:ti,ab,kw OR expensive:ti,ab,kw OR expenditure:ti,ab,kw OR expenditures:ti,ab,kw OR pay:ti,ab,kw OR paid:ti,ab,kw OR price:ti,ab,kw OR budget:ti,ab,kw OR sustainability:ti,ab,kw OR sustained:ti,ab,kw OR sustainable:ti,ab,kw OR appropriateness:ti,ab,kw OR appropriate:ti,ab,kw OR suitable:ti,ab,kw OR suitability:ti,ab,kw OR suited:ti,ab,kw OR relevance:ti,ab,kw OR relevant:ti,ab,kw OR penetration:ti,ab,kw OR penetrated:ti,ab,kw OR diffusion:ti,ab,kw OR adoption:ti,ab,kw OR adopted:ti,ab,kw OR implementation science:ti,ab,kw OR implementation research:ti,ab,kw OR dissemination research:ti,ab,kw OR implementation outcom*:ti,ab,kw

#3: “mental health” OR “wellbeing” OR “psychosocial wellbeing” OR “mental health intervention” OR “scalable intervention” OR “psychological”

#4: #1 AND #2 AND #3

d. SCOPUS

(TITLE-ABS-KEY("problem management plus" OR "step-by-step" OR "group problem management plus" OR "PM+" OR "gPM+")) AND (TITLE-ABS-KEY("acceptability" OR "acceptable" OR "feasibility" OR "feasible" OR "fidelity" OR "adherent" OR "adherence" OR "accuracy" OR "accurate" OR "implementation cost*" OR "cost" OR "economic*" OR "expense" OR "expensive" OR "expenditure" OR "expenditures" OR "pay" OR "paid" OR "price" OR "budget" OR "sustainability" OR "sustained" OR "sustainable" OR "appropriateness" OR "appropriate" OR "suitable" OR "suitability" OR "suited" OR "relevance" OR "relevant" OR "penetration" OR "penetrated" OR "diffusion" OR "adoption" OR "adopted" OR "implementation science" OR "implementation research" OR "dissemination research" OR "implementation outcom*")) AND (TITLE-ABS-KEY("mental health" OR "wellbeing" OR "psychosocial wellbeing" OR "mental health intervention" OR "scalable intervention" OR "psychological"))

3. **Appendix C: Full references**

C1: Included studies

Abi Hana, R., Abi Ramia, J., Burchert, S., Carswell, K., Cuijpers, P., Heim, E., ... & Smit, F. (2024). Cost-Effectiveness of Digital Mental Health Versus Usual Care During Humanitarian Crises in Lebanon: Pragmatic Randomized Trial. *JMIR Mental Health*, *11*(1), e55544.

Abi Ramia, J., Abi Hana, R., Noun, P., Cuijpers, P., Carswell, K., Van't Hof, E., ... & El Chammay, R. (2024). Feasibility and uptake of a digital mental health intervention for depression among Lebanese and Syrian displaced people in Lebanon: a qualitative study. *Frontiers in public health*, *11*, 1293187.

Acarturk, C., Uygun, E., Ilkkursun, Z., Yurtbakan, T., Kurt, G., Adam-Troian, J., ... & Fuhr, D. C. (2022). Group problem management plus (PM+) to decrease psychological distress among Syrian refugees in Turkey: a pilot randomised controlled trial. *BMC psychiatry*, *22*(1), 8.

Akhtar, A., Giardinelli, L., Bawaneh, A., Awwad, M., Al-Hayek, H., Whitney, C., ... & Bryant, R. (2021). Feasibility trial of a scalable transdiagnostic group psychological intervention for Syrians residing in a refugee camp. *European Journal of Psychotraumatology*, *12*(1), 1932295.

Bryant, R. A., Bawaneh, A., Awwad, M., Al-Hayek, H., Giardinelli, L., Whitney, C., ... & Akhtar, A. (2022). Twelve-month follow-up of a randomised clinical trial of a brief group psychological intervention for common mental disorders in Syrian refugees in Jordan. *Epidemiology and Psychiatric Sciences*, *31*, e81.

Burchert, S., Alkneme, M. S., Alsaod, A., Cuijpers, P., Heim, E., Hessling, J., ... & STRENGTHS Consortium. (2024). Effects of a self-guided digital mental health self-help intervention for Syrian refugees in Egypt: A pragmatic randomized controlled trial. *PLoS medicine*, *21*(9), e1004460.

Burchert, S., Alkneme, M. S., Bird, M., Carswell, K., Cuijpers, P., Hansen, P., ... & Knaevelsrud, C. (2019). User-centered app adaptation of a low-intensity e-mental health intervention for Syrian refugees. *Frontiers in psychiatry*, *9*, 663.

Cuijpers, P., Heim, E., Abi Ramia, J., Burchert, S., Carswell, K., Cornelisz, I., ... & El Chammay, R. (2022). Guided digital health intervention for depression in Lebanon: randomised trial. *Evidence Based Mental Health*, *25*(e1).

Dawson, K. S., Schafer, A., Anjuri, D., Ndogoni, L., Musyoki, C., Sijbrandij, M., ... & Bryant, R. A. (2016). Feasibility trial of a scalable psychological intervention for women affected by urban adversity and gender-based violence in Nairobi. *BMC psychiatry*, *16*(1), 410.

Dozio, E., Dill, A. S., & Bizouerne, C. (2021). Problem Management Plus adapted for group use to improve mental health in a war-affected population in the Central African Republic. *Intervention Journal of Mental Health and Psychosocial Support in Conflict Affected Areas*, *19*(1), 91-100.

Greene, M. C., Castellar, D., Sangraula, M., Camargo, N., Diaz, J., Meriño, V ., ... & Brown, A. (2024). Comparing implementation strategies for training and supervising nonspecialists in Group Problem Management Plus: A hybrid effectiveness-implementation trial in Colombia. *Cambridge Prisms: Global Mental Health*, *11*, e90.

Hamdani, S. U., Rahman, A., Wang, D., Chen, T., van Ommeren, M., Chisholm, D., & Farooq, S. (2020). Cost-effectiveness of WHO Problem Management Plus for adults with mood and anxiety disorders in a post-conflict area of Pakistan: randomised controlled trial. *The British Journal of Psychiatry*, *217*(5), 623-629.

Heim, E., Abi Ramia, J., Abi Hana, R., Burchert, S., Carswell, K., Cornelisz, I., ... & Van't Hof, E. (2021). Step-by-step: feasibility randomised controlled trial of a mobile-based intervention for depression among populations affected by adversity in Lebanon. *Internet interventions*, *24*, 100380.

Hussain, B., Khalily, M. T., Waqas, A., Rahman, A., Angelakis, I., Nisar, A., ... & Akhtar, T. (2025). Acceptability and efficacy of the culturally adapted problem management plus intervention for people with disability in Pakistan: a pilot cluster randomized controlled trial. *Frontiers in Psychiatry*, *15*, 1413809.

Kangwana, B., Mutahi, J., & Kumar, M. (2024). Experiences of integrating a psychological intervention into a youth-led empowerment program targeting out-of-school adolescents, in urban informal settlements in Kenya: A qualitative study. *Plos one*, *19*(4), e0300463.

Khan, M. N., Hamdani, S. U., Chiumento, A., Dawson, K., Bryant, R. A., Sijbrandij, M., ... & Rahman, A. (2019). Evaluating feasibility and acceptability of a group WHO trans-diagnostic intervention for women with common mental disorders in rural Pakistan: a cluster randomised controlled feasibility trial. *Epidemiology and psychiatric sciences*, *28*(1), 77-87.

McBain, R. K., Mwale, O., Mpinga, K., Kamwiyo, M., Kayira, W., Ruderman, T., ... & Wagner, G. J. (2024). Effectiveness, cost-effectiveness, and positive externalities of integrated chronic care for adults with major depressive disorder in Malawi (IC3D): a stepped-wedge, cluster-randomised, controlled trial. *The Lancet*, *404*(10465), 1823-1834.

Miller-Suchet, L., Camargo, N., Sangraula, M., Castellar, D., Diaz, J., Meriño, V ., ... & Greene, M. C. (2024). Comparing mediators and moderators of mental health outcomes from the implementation of Group Problem Management Plus (PM+) among Venezuelan refugees and migrants and Colombian returnees in Northern Colombia. *International Journal of Environmental Research and Public Health*, *21*(5), 527.

Mwale, O., Kasambara, K., Houde, A., Mpinga, K., Kayira, W., Harawa, M., ... & McBain, R. K. (2025). Patient perspectives on group problem management plus for adults with major depressive disorder in rural Malawi. *Global Health Action*, *18*(1), 2500785.

Nakkash, R., Fares, M., Tleis, M., Mugharbil, S., Antaby, M., Al Masri, H., ... & Afifi, R. (2024). Power sharing in community-engaged research with Syrian refugees in Lebanon: Using community engagement to shape intervention fit to context. *SSM-Mental Health*, *6*, 100358.

Perera, C., Aldamman, K., Hansen, M., Haahr-Pedersen, I., Caballero-Bernal, J., Caldas-Castañeda, O. N., ... & Vallières, F. (2022). A brief psychological intervention for improving the mental health of Venezuelan migrants and refugees: A mixed-methods study. *SSM-Mental Health*, *2*, 100109.

Sangraula, M., Turner, E. L., Luitel, N. P., Van‘t Hof, E., Shrestha, P., Ghimire, R., ... & Jordans, M. J. D. (2020). Feasibility of Group Problem Management Plus (PM+) to improve mental health and functioning of adults in earthquake-affected communities in Nepal. *Epidemiology and Psychiatric Sciences*, *29*, e130.

Van't Hof, E., Dawson, K. S., Schafer, A., Chiumento, A., Shehadeh, M. H., Sijbrandij, M., ... & van Ommeren, M. (2018). A qualitative evaluation of a brief multicomponent intervention provided by lay health workers for women affected by adversity in urban Kenya. Global Mental Health, 5, e6.

C2: Studies ongoing

Helova, A., Onono, M., Ogolla-Onyando, M., Ouma, E., Imran, R., Beres, L. K., Hampanda, K.,

Owuor, K., Szychowski, J. M., Ongeri, L., Abuogi, L. L., & Turan, J. M. (2025). Evaluation of risk stratification and problem management plus (PM+) for pregnant women with HIV in Kenya (Tatua study): Protocol paper. Contemporary Clinical Trials, 151, 107838. https://doi.org/10.1016/j.cct.2025.107838

Keyan, D., Habashneh, R., Akhtar, A., El-Dardery, H., Faroun, M., Abualhaija, A., Aqel, I. S., Dardas, L. A., & Bryant, R. (2024). Evaluating a stepped care model of psychological support for adults affected by adversity: Study protocol for a randomised controlled trial in Jordan. BMJ Open, 14(2), e078091. https://doi.org/10.1136/bmjopen-2023-078091

Logie, C. H., Okumu, M., Kortenaar, J.L., Gittings, L., Khan, N., Hakiza, R., Kibuuka Musoke, D.,Nakitende, A., Katisi, B., Kyambadde, P., Khan, T., Lester, R., & Mbuagbaw, L. (2022). Mobile Health-Supported Virtual Reality and Group Problem Management Plus: Protocol for a Cluster Randomized Trial Among Urban Refugee and Displaced Youth in Kampala, Uganda (Tushirikiane4MH, Supporting Each Other for Mental Health). JMIR Research Protocols, 11(12), e42342. https://doi.org/10.2196/42342

Massazza, A., Fuhr, D., Bogdanov, S., Tol, W., Roberts, B., Nadkarni, A., Akudrabo, G., Andersen, L., Dumchev, K., Karachevskyy, A., Kinyanda, E., Koss, K., Moore, Q., & May, C. (2022). A Complex Intervention for Alcohol Misuse Among Conflict-Affected Populations in Uganda and Ukraine: Study Protocol for the Qualitative Components in the CHANGE Trial. International Journal of Qualitative Methods, 20, 160940692110630. https://doi.org/10.1177/16094069211063031

Smith, S. L., Nyirandagijimana, B., Hakizimana, J., Levy, R. P., Bienvenu, R., Uwamwezi, A., Hakizimfura, O., Uwimana, E., Kundu, P., Mpanumusingo, E., Nshimyiryo, A., Rusangwa, C., Kateera, F., Mukasakindi,

H., & Raviola, G. (2021). Evaluating the delivery of Problem Management Plus in primary care settings in rural Rwanda: A study protocol using a pragmatic randomised hybrid type 1 effectiveness-implementation design. BMJ Open, 11(12), e054630. https://doi.org/10.1136/bmjopen-2021-054630

van der Boor, C. F., Taban, D., Tol, W. A., Akellot, J., Neuman, M., Weiss, H. A., Greco, G., Vassall, A., May, C., Nadkarni, A., Kinyanda, E., Roberts, B., & Fuhr, D. C. (2024). Effectiveness and cost-effectiveness of a transdiagnostic intervention for alcohol misuse and psychological distress in humanitarian settings: Study protocol for a randomised controlled trial in Uganda. Trials, 25, 148. https://doi.org/10.1186/s13063-024-07980-7.

C3: Excluded studies

Reference	Reason for exclusion
Akhtar, A., Engels, M. H., Bawaneh, A., Bird, M., Bryant, R., Cuijpers, P., Hansen, P., Al-Hayek, H., Ilkkursun, Z., Kurt, G., Sijbrandij, M., Underhill, J., Acarturk, C., & Consortium, O. behalf of the S. (2021). Cultural Adaptation of a Low-Intensity Group Psychological Intervention for Syrian Refugees. *Intervention Journal of Mental Health and Psychosocial Support in Conflict Affected Areas* , *19* (1), 48. https://doi.org/10.4103/INTV.INTV_38_20	Wrong outcome
Akhtar, A., Giardinelli, L., Bawaneh, A., Awwad, M., Naser, H., Whitney, C., Jordans, M. J. D., Sijbrandij, M., Bryant, R. A., & on behalf of the STRENGTHS Consortium. (2020). Group problem management plus (gPM+) in the treatment of common mental disorders in Syrian refugees in a Jordanian camp: Study protocol for a randomized controlled trial. *BMC Public Health* , *20* (1), 390. https://doi.org/10.1186/s12889-020-08463-5	Wrong outcome
Chiumento, A., Hamdani, S. U., Khan, M. N., Dawson, K., Bryant, R. A., Sijbrandij, M., Nazir, H., Akhtar, P., Masood, A., Wang, D., van Ommeren, M., & Rahman, A. (2017). Evaluating effectiveness and cost-effectiveness of a group psychological intervention using cognitive behavioural strategies for women with common mental disorders in conflict-affected rural Pakistan: Study protocol for a randomised controlled trial. *Trials* , *18* (1), 190. https://doi.org/10.1186/s13063-017-1905-8	Pilot/Protocol of already included study
Fink, G., Melero-Dominguez, M., Chembe, M., Vernisy-Romero, D. de, Tembo, T., Billima, T., Paul, R., Alegria, M., Parkerson, D., Rockers, P. C., Banda, Z., Lungu, G., Sikazwe, D., & Falgas-Bague, I. (2024). Feasibility and acceptability of the Problem Management for Moms programme for improving maternal mental health in Zambia: An open-label trial. *The Lancet Psychiatry* , *11* (12), 965–974. https://doi.org/10.1016/S2215-0366(24)00256-6	Wrong setting
Graaff, A. M. de, Cuijpers, P., McDaid, D., Park, A., Woodward, A., Bryant, R. A., Fuhr, D. C., Kieft, B., Minkenberg, E., & Sijbrandij, M. (2020). Peer-provided Problem Management Plus (PM+) for adult Syrian refugees: A pilot randomised controlled trial on effectiveness and cost-effectiveness. *Epidemiology and Psychiatric Sciences* , *29* , e162. https://doi.org/10.1017/S2045796020000724	Wrong setting
Hamdani, S. U., Ahmed, Z., Sijbrandij, M., Nazir, H., Masood, A., Akhtar, P., Amin, H., Bryant, R. A., Dawson, K., van Ommeren, M., Rahman, A., & Minhas, F. A. (2017). Problem Management Plus (PM+) in the management of common mental disorders in a specialized mental healthcare facility in Pakistan; study protocol for a randomized controlled trial. *International Journal of Mental Health Systems* , *11* , 40. https://doi.org/10.1186/s13033-017-0147-1	Pilot/Protocol of already included study
Jordans, M. J. D., Kohrt, B. A., Sangraula, M., Turner, E. L., Wang, X., Shrestha, P., Ghimire, R., Van't Hof, E., Bryant, R. A., Dawson, K. S., Marahatta, K., Luitel, N. P., & van Ommeren, M. (2021). Effectiveness of Group Problem Management Plus, a brief psychological intervention for adults affected by humanitarian disasters in Nepal: A cluster randomized controlled trial. *PLoS Medicine* , *18* (6), e1003621. https://doi.org/10.1371/journal.pmed.1003621	Wrong outcome
McBain, R. K., Mwale, O., Ruderman, T., Kayira, W., Connolly, E., Chalamanda, M., Kachimanga, C., Khongo, B. D., Wilson, J., Wroe, E., Raviola, G., Smith, S., Coleman, S., Kelly, K., Houde, A., Tebeka, M. G., Watson, S., Kulisewa, K., Udedi, M., & Wagner, G. (2021). Stepped care for depression at integrated chronic care centers (IC3) in Malawi: Study protocol for a stepped-wedge cluster randomized controlled trial. *Trials* , *22* (1), 630. https://doi.org/10.1186/s13063-021-05601-1	Pilot/Protocol of already included study
Mwale, O., Kasambala, C., Houde, A., Mpinga, K., Kayira, W., Harawa, M., Kamwiyo, M., Isaacs, R., Nhlema, B., Ruderman, T., Liwimbi, O., Udedi, M., Kelly, K., & McBain, R. K. (2025). Patient perspectives on group problem management plus for adults with major depressive disorder in rural Malawi. *Global Health Action* , *18* (1), 2500785. https://doi.org/10.1080/16549716.2025.2500785	Wrong setting
Sangraula, M., Greene, M. C., Castellar, D., Flechas de la Hoz, J. C., Diaz, J., Merino, V., Miller-Suchet, L., Cristobal, M., Coneo, A. M. C., Morales, L., Venegas, M., Gonzalez, A. G., DeLuca, M., Uribe, M., Romero, S. A., Ferrer, M., Snider, L., Marsch, L. A., Espinel, Z., … Brown, A. D. (2023). Protocol for a Randomized Hybrid Type 2 Trial on the Implementation of Group Problem Management Plus (PM+) for Venezuelan Women Refugees and Migrants in Colombia. *Intervention Journal of Mental Health and Psychosocial Support in Conflict Affected Areas* , *21* (2), 154. https://doi.org/10.4103/intv.intv_4_23	Pilot/Protocol of already included study
Sangraula, M., Hof, E., Luitel, N., Turner, E., Marahatta, K., Nakao, J., Ommeren, M., Jordans, M., & Kohrt, B. (2018). Protocol for a feasibility study of group-based focused psychosocial support to improve the psychosocial well-being and functioning of adults affected by humanitarian crises in Nepal: Group Problem Management Plus (PM+). *Pilot and Feasibility Studies* , *4* . https://doi.org/10.1186/s40814-018-0315-3	Pilot/Protocol of already included study
Sapkota, D., Baird, K., Saito, A., Rijal, P., Pokharel, R., & Anderson, D. (2019). Counselling-based psychosocial intervention to improve the mental health of abused pregnant women: A protocol for randomised controlled feasibility trial in a tertiary hospital in eastern Nepal. *BMJ Open* , *9* . https://doi.org/10.1136/bmjopen-2018-027436	Wrong intervention
Sijbrandij, M., Farooq, S., Bryant, R. A., Dawson, K., Hamdani, S. U., Chiumento, A., Minhas, F., Saeed, K., Rahman, A., & van Ommeren, M. (2015). Problem Management Plus (PM+) for common mental disorders in a humanitarian setting in Pakistan; study protocol for a randomised controlled trial (RCT). *BMC Psychiatry* , *15* , 232. https://doi.org/10.1186/s12888-015-0602-y	Pilot/Protocol of already included study
Spaaij, J., Kiselev, N., Berger, C., Bryant, R. A., Cuijpers, P., de Graaff, A. M., Fuhr, D. C., Hemmo, M., McDaid, D., Moergeli, H., Park, A.-L., Pfaltz, M. C., Schick, M., Schnyder, U., Wenger, A., Sijbrandij, M., & Morina, N. (2022). Feasibility and acceptability of Problem Management Plus (PM+) among Syrian refugees and asylum seekers in Switzerland: A mixed-method pilot randomized controlled trial. *European Journal of Psychotraumatology* , *13* (1), 2002027. https://doi.org/10.1080/20008198.2021.2002027	Wrong setting
Tay, A. K., Mung, H. K., Miah, M. A. A., Balasundaram, S., Ventevogel, P., Badrudduza, M., Khan, S., Morgan, K., Rees, S., Mohsin, M., & Silove, D. (2020). An Integrative Adapt Therapy for common mental health symptoms and adaptive stress amongst Rohingya, Chin, and Kachin refugees living in Malaysia: A randomized controlled trial. *PLoS Medicine* , *17* (3), e1003073. https://doi.org/10.1371/journal.pmed.1003073	Wrong intervention
van't Hof, E., Sangraula, M., Luitel, N. P., Turner, E. L., Marahatta, K., van Ommeren, M., Shrestha, P., Bryant, R., Kohrt, B. A., & Jordans, M. J. D. (2020). Effectiveness of Group Problem Management Plus (Group-PM+) for adults affected by humanitarian crises in Nepal: Study protocol for a cluster randomized controlled trial. *Trials* , *21* (1), 343. https://doi.org/10.1186/s13063-020-04263-9	Pilot/Protocol of already included study
Wambua, G. N., Stein, A., Seedat, S., Sijbrandij, M., Baisley, K., Shahmanesh, M., Seeley, J., & Ngwenya, N. (2024). Adaptation and feasibility of WHO PM+ for adolescents living with HIV in KwaZulu-Natal Province, South Africa: An implementation feasibility study protocol. *BMJ Open* , *14* (7), e088992. https://doi.org/10.1136/bmjopen-2024-088992	Wrong setting

4. Appendix D: Study by study table

Study	Country	Population	Intervention	Setting	MMAT rating (out of 5)	Acceptability	Feasibility	Fidelity	Implementation costs	Sustainability	Appropriateness
Abi Hana et al., 2024	Lebanon	Community residents	Step-by-Step	Crisis or Conflict Settings	3		The study faced a high and expected dropout rate, which may have influenced the outcomes.		The SbS intervention cost $26 per user (vs. $0.01 for EUC), with total social costs lower for SbS ($393) than EUC ($443) per participant.		
Abi Ramia et al., 2023	Lebanon	Key Informant	Step-by-Step	Refugee or Displacement Settings	4	Key informants agreed the SbS intervention was beneficial, practical, and culturally appropriate in Lebanon.	RCTs reported high dropout rates, particularly among Lebanese (65.1%) and Syrians (46.2%).				
Acarturk et al., 2022	Turkey	Displaced/Migrant Adults	gPM+ and other therapy	Refugee or Displacement Settings	4	Syrian refugees viewed gPM+ positively for its group sharing, useful strategies, and facilitator support despite participation barriers.	Retention in gPM+ was strong, with 75% of participants completing the program.	Fidelity assessments found 80% of core gPM+ components were delivered well, with 20% needing improvement.			
Akhtar et al., 2021	Jordan	Displaced/Migrant Adults	gPM+ and other therapy	Refugee or Displacement Settings	5		GroupPM+ showed good cultural acceptance, with most participants who started the intervention attending three or more sessions.				

Study	Country	Population	Intervention	Setting	MMAT rating (out of 5)	Acceptability	Feasibility	Fidelity	Implementation costs	Sustainability	Appropriateness
Bryant et al., 2022	Jordan	Displaced/Migrant Adults	gPM+ and other therapy	Refugee or Displacement Settings	4		At 12 months, 74.9% of participants were retained, with higher retention in the EUC condition than in gPM+ (79.6% vs. 70.1%).				
Burchert et al., 2019	Egypt	Displaced/Migrant Adults	Step-by-Step	Refugee or Displacement Settings	2	While many trusted the program's privacy, some Syrians struggled with acceptance of psychological help and app-based delivery.					
Burchert et al., 2024	Egypt	Displaced/Migrant Adults	Step-by-Step	Refugee or Displacement Settings	3		168/538 drop-out				
Cuijpers et al., 2022	Lebanon	Community residents	Step-by-Step	Crisis or Conflict Settings	2	Over 90% of participants were satisfied and would return to the program if in need of help again.	Although initial attendance was 60.1%, only 19.3% completed all sessions, reflecting high dropout despite some reporting high completion.	Fidelity checks showed only 6% minor deviations from the treatment protocol.			
Dawson et al., 2016	Kenya	Adults with Elevated Distress	PM+ and other therapy	Urban/Non-Refugee Settings	3		PM+ had higher post-assessment participation than ETAU (86% vs. 71%), showing CHWs in Kenya can effectively deliver the intervention.	Community Health Workers (CHWs) were able to deliver PM+ effectively after brief training and supervision.			

Study	Country	Population	Intervention	Setting	MMAT rating (out of 5)	Acceptability	Feasibility	Fidelity	Implementation costs	Sustainability	Appropriateness
Dozio, Dill and Bizouerne, 2021	Central African Republic	Community residents	gPM+	Crisis or Conflict Settings	3	Most participants gained knowledge, felt more socially connected, relaxed, and experienced reduced isolation and stress.					
Greene et al., 2024	Colombia	Adults in Care Settings	gPM+	Refugee or Displacement Settings	5	Participants experienced improved communication and problem-solving but faced barriers like illness and family duties.	Engagement and completion rates varied, with higher retention in groups receiving specialized technical support (69.1% vs. 23.7%).	Higher fidelity to session content was observed in the nonspecialized support condition (78%) than in the specialized condition (64%).	Total study cost was $67,393, with per-arm costs varying across components from $295 to $31,728.	Facilitators and participants supported continued community delivery of gPM+, emphasizing the need for certification and organizational backing to enhance sustainability and trust.	
Hamdani et al., 2020	Pakistan	Adults with Elevated Distress	PM+ and other therapy	Crisis or Conflict Settings	2				Delivering PM+ cost $163.14 per participant with international trainers (vs. $35.04 with local trainers), with local delivery showing greater cost-effectiveness.		
Heim et al., 2021	Lebanon	Displaced/Migrant Adults	Step-by-Step	Refugee or Displacement Settings	2	Participants found the intervention empowering and relaxing, especially in contexts with high mental health stigma.	Dropout was higher in the control group than the intervention group, mainly due to technical issues and life challenges.				

Study	Country	Population	Intervention	Setting	MMAT rating (out of 5)	Acceptability	Feasibility	Fidelity	Implementation costs	Sustainability	Appropriateness
Hussain et al., 2025	Pakistan	Adults in Care Settings	PM+	Urban/Non-Refugee Settings	5	The qualitative interviews regarding the intervention revealed its high acceptability among participants, primarily attributed to the development of cognitive and behavioural coping skills.	All the participants who were randomised, completed the study (n = 148) indicating anexcellent retention rate (100%).	Not explicitly quantified as a fidelity score, but the text indicates efforts were made to "maintain protocol fidelity" and "ensuring the adherence to the IA-PM+ protocols".			
Kangwana, Muthani and Kumar, 2023	Kenya	Adolescents	PM+	Urban/Non-Refugee Settings	5	Parents, adolescents, and mentors saw mental health improvements and gained skills to manage personal challenges.		Mentors adhered well to core principles of delivering psychological interventions.			
Khan et al., 2019	Pakistan	Health workers	gPM+ and other therapy	Crisis or Conflict Settings	5	Group PM+ was praised for improving psychological well-being, emotional relief, and enhancing social connections.	61% of participants attended all five sessions, and 94% were retained, indicating strong adherence and community acceptance.				
McBain et al., 2024	Malawi	Adults in Care Settings	PM+	Urban/Non-Refugee Settings	4		89 (18%) of 487 participants never initiated treatment.	Not explicitly quantified in the provided snippets, but the method was in place "To bolster protocol fidelity"	The intervention improved cost-effectiveness by ~30%, costing \$98 per person with an ICER of \$481 per DALY averted—below the \$645 threshold		

									in 80% of simulations.		
**Study**	**Country**	**Population**	**Intervention**	**Setting**	**MMAT rating (out of 5)**	**Acceptability**	**Feasibility**	**Fidelity**	**Implementation costs**	**Sustainability**	**Appropriateness**
Miller-Such et et al., 2024	Colombia	Displaced/Migrant Adults	gPM+	Refugee or Displacement Settings	5	Participants gained additional coping tools like better communication, patience, and emotional understanding beyond core PM+ skills.					
Mwale et al., 2025	Malawi	Community residents	PM+	Urban/Non-Refugee Settings	5	Overall, all participants expressed acceptance of PM+ as a treatment option.	The preponderance (94%) of key informants attended all five PM+ sessions, while remaining individuals (6%) attended 3–4 PM+ sessions.	Not explicitly reported in the paper's results			
Nakkash et al., 2024	Lebanon	Displaced/Migrant Adults	PM+	Refugee or Displacement Settings	5						Community-engaged research helped tailor the PM+ intervention to the Syrian refugee community by incorporating CAC members' culturally relevant suggestions into the study design, tools, and approach.
Perera et al., 2022	Colombia	Displaced/Migrant Adults	PM+	Refugee or Displacement Settings	4	PM+ sessions were viewed as safe and supportive spaces, with volunteers seen as well-prepared and professional.	The study had a low dropout rate (5.3%), suggesting strong participant satisfaction and high retention.				

Study	Country	Population	Intervention	Setting	MMAT rating (out of 5)	Acceptability	Feasibility	Fidelity	Implementation costs	Sustainability	Appropriateness
Sangraula et al., 2020	Nepal	Community residents	gPM+ and other therapy	Crisis or Conflict Settings	3	Participants found PM+ a safe space for sharing distress, with volunteers seen as professional and supportive.	This Group PM+ trial, including males for the first time, showed high feasibility and excellent retention (97.5%).	All Group PM+ facilitators achieved at least 75% fidelity across all five sessions.			
van't Hof et al., 2018	Kenya	Key informants	PM+	Urban/Non-Refugee Settings	5	PM+ was well accepted, with participants and CHVs reporting personal growth, stress management, and improved well-being.		Community Health Volunteers (CHVs) were seen as effective delivery agents for the intervention.		While CHVs are well-positioned to deliver PM+ within Kenya's PHC system, sustainability is threatened by limited funding and challenges in CHV selection, training, supervision, and compensation.	
